# The 150 most important questions in cancer research and clinical oncology series: questions 50–56

**DOI:** 10.1186/s40880-017-0236-1

**Published:** 2017-08-29

**Authors:** Chinese Journal of Cancer

**Affiliations:** Sun Yat-sen University Cancer Center, Guangzhou, 510060 Guangdong P. R. China

**Keywords:** Bone marrow niche, Tipping point during cancer progression, Network biomarkers, Targeted chemotherapeutics, Somatic genome alteration, Immune evasion mechanism, Sarcomatoid carcinoma

## Abstract

Since the beginning of 2017, *Chinese Journal of Cancer* has published a series of important questions in cancer research and clinical oncology, which sparkle diverse thoughts, interesting communications, and potential collaborations among researchers all over the world. In this article, seven more questions are presented as followed. Question 50. When tumor cells spread from primary site to distant sites, are they required to be “trained” or “armed” in the bone marrow niche prior to colonizing soft tissues? Question 51. Are there tipping points during cancer progression which can be identified for manipulation? Question 52. Can we replace molecular biomarkers by network biomarkers? Question 53. Are conventional inhibitors of key cellular processes such as cell proliferation and differentiation more effective than targeted chemotherapeutics that antagonize the downstream cell signaling network via cell-surface receptors such as epidermal growth factor receptor (EGFR), vascular endothelial growth factor receptor (VEGFR) and c-Met, or intracellular receptors such as androgen receptor (AR) and estrogen receptor (ER), by drugs like erlotinib, sunitinib and cabozantinib, or enzalutamide and tomoxifen? Question 54. How can we robustly identify the candidate causal event of somatic genome alteration (SGA) by using computational approach? Question 55. How can we systematically reveal the immune evasion mechanism exploited by each tumor and utilize such information to guide targeted therapy to restore immune sensitivity? Question 56. Can the nasopharyngeal carcinoma (NPC) patients with sarcomatoid carcinoma (SC) subtype benefit from more specific targeted therapy?

## Text

To accelerate our endeavors to overcome cancer, *Chinese Journal of Cancer* has launched a program of publishing 150 most important questions in cancer research and clinical oncology [1]. Since the beginning of 2017, *Chinese Journal of Cancer* has published a series of important questions in cancer research and clinical oncology [2–8], which sparkle diverse thoughts, interesting communications, and potential collaborations among researchers all over the world. In this article, Questions 50–56 are selected and presented. This program of collecting and publishing the key questions is still ongoing. Please send your thoughtful questions to Ms. Ji Ruan via email: ruanji@sysucc.org.cn.

## Question 50: When tumor cells spread from primary site to distant sites, are they required to be “trained” or “armed” in the bone marrow niche prior to colonizing soft tissues?

### Background and implications

The progression of prostate and breast cancers is often characterized by their transition from a hormone-sensitive to a hormone-independent state. A small fraction of the cells, both hormone-sensitive cells and hormone-resistant cells, is believed to undergo epithelial-to-mesenchymal transition at the primary site, conferring increased ability to invade, migrate, and metastasize. Ultimately, these disseminated tumor cells appear in the blood compartment as circulating tumor cells, gain access to and colonize the bone. Clinically, when patients are treated with effective bone-targeted therapeutic agents, increased dissemination of prostate and breast cancers occurs in the liver, brain, and lung. The bone is a nutrient-rich environment with ample growth factors, chemokines, and cytokines that could “train” or “arm” migrating tumor cells seeded in the bone from the circulation to undergo further genetic/epigenetic changes in the bone microenvironment that prepare these cells for their subsequent journey toward soft tissues such as the liver, brain, and lung. This idea is supported by laboratory studies in which delivery of tumor cells to the bone often results in their dissemination to soft tissues, and in which certain soluble factors in the bone were shown to confer the specific ability of homing to soft tissues by cancer cells. Effective therapeutics targeting the lethal progression of cancer to soft tissues may require the ability to untangle cancer cell interactions with the host bone marrow niche.

#### Submitter

Haiyen E. Zhau.

#### Affiliation and email

Department of Medicine, Samuel Oschin Comprehensive Cancer Institute, Cedars-Sinai Medical Center, Los Angeles, CA, USA.

haiyen.zhau@cshs.org.

## Question 51: Are there tipping points during cancer progression which can be identified for manipulation?

### Background and implication

Considerable evidence suggests that, during cancer progression, the deteriorations are not necessarily smooth but are abrupt, and may cause an irreversible transition from one state to another at a tipping point. I hypothesize that, rather than the expression alterations of individual driver genes, a tipping point could be the associative alterations among the driver genes, i.e., the alterations of a molecular network consisting of subtle expression alterations of multiple genes connected to each others. If we can prove this hypothesis and further quantify the tipping point as well as its dynamic network biomarkers, we will then open a new door for cancer prevention and treatment.

#### Submitter

Luonan Chen.

#### Affiliation and email

Key Laboratory of Systems Biology, Shanghai Institutes for Biological Sciences, Chinese Academy of Sciences, Shanghai, China.

lnchen@sibs.ac.cn.

## Question 52: Can we replace molecular biomarkers by network biomarkers?

### Background and implication

Molecular biomarkers are mainly represented by the concentrations, which are changeable with different physiological conditions and are generally unstable. In contrast, a molecular network of the examined tissues can reliably and stably reflect the physiologic and/or pathologic conditions, which is a better mean for disease diagnosis and prognostic prediction. However, it is challenging to characterize a molecular network in one single tissue sample using traditional analytical approach. A revolutionary comprehensive approach should be developed to identify the associative network of molecules in a single tissue sample, replacing the traditionally individual biomarker detection. This research direction could have great implications on both biology and medicine.

#### Submitter

Luonan Chen.

#### Affiliation and email

Key Laboratory of Systems Biology, Shanghai Institutes for Biological Sciences, Chinese Academy of Sciences, Shanghai, China.

lnchen@sibs.ac.cn.

## Question 53: Are conventional inhibitors of key cellular processes such as cell proliferation and differentiation more effective than targeted chemotherapeutics that antagonize the downstream cell signaling network via cell-surface receptors such as epidermal growth factor receptor (EGFR), vascular endothelial growth factor receptor (VEGFR) and c-Met, or intracellular receptors such as androgen receptor (AR) and estrogen receptor (ER), by drugs like erlotinib, sunitinib and cabozantinib, or enzalutamide and tomoxifen?

### Background and implications

In the past decade, despite significant progress in understanding the inter- and intra-cellular signaling network, the mutational landscape in human cancers, and the development of receptor and receptor tyrosine kinase-based targeted strategies, the painful reality we face is that there is no improvement in the 5-year survival rate of cancer patients with localized (no metastasis), regional (with early lymph node metastasis), and distant (disseminated metastases to the bone, liver, or lung) disease [9]. These statistics are supported by observations in the clinic where patients treated with targeted therapeutics often experience a short-term survival advantage in months. These results are in sharp contrast to our early chemotherapeutic experience in which we observed the “cure” of men with widely disseminated germ-cell and stem-cell testicular cancers by cytotoxic agents such as cisplatin that intercalate cellular DNA and prevent further replication and differentiation. This raises the important question of whether we have ignored the power of agents developed to target the final common pathway that collectively controls the fundamental hallmark of cancer, its uncontrolled cell proliferation and failed differentiation, and apoptosis. To improve our dismal accomplishments of curing cancer patients with deadly cancer metastases over the past decade, should we begin to change our strategies and repurpose older drugs shown to cure cancer, improving the delivery of these drugs by newer and more effective delivery vehicles, or seek to develop more potent cytotoxic drugs that kill cancer cells by blocking proliferation and differentiation rather than the current approach focusing on the development of targeted receptor-based therapeutics?

#### Submitter

Leland W. K. Chung.

#### Affiliation and email

Department of Medicine and Surgery, Cedars-Sinai Medical Center, Los Angeles, CA, USA.

leland.chung@cshs.org.

## Questions 54: How can we robustly identify the candidate causal event of somatic genome alteration (SGA) by using computational approach?

### Background and implication

Cancer is a genomic disease caused by somatic genome alterations (SGAs) that perturb the function of proteins, which in turn disturb the normally well-controlled cellular signaling system. It is not uncommon that a cancer cell hosts over hundreds to thousands of SGAs, and the foremost task of precision oncology is to decide which SGAs are drivers in an individual tumor so that appropriate therapeutics can be employed to correct the aberrations resulting from these driver SGAs. Our current knowledge of cancer driver genes is incomplete, thus a simple look up whether the known driver genes [10, 11] are among SGAs of a tumor is not sufficient; furthermore, not all mutations in a known cancer driver are necessarily driving events. The unanswered questions are how to determine whether an SGA event in a given tumor contributes to oncogenic processes of the tumor, of which the state of affected oncogenic processes can be molecular phenotypic changes, such as transcriptomic, proteomic, and metabolomic changes. A computational approach should therefore be developed to robustly infer the causal relationships between SGA events and molecular phenotypic changes to discover the causative SGA events in tumors. Answering these questions will not only discover candidate drivers but also reveal the functional impacts of the drivers in an individual tumor. Such information sheds light on the disease mechanism of the tumor and can be used to guide precision oncology.

#### Submitter

Xinghua Lu.

#### Affiliation and email

Department of Biomedical Informatics, University of Pittsburgh, Pittsburgh, PA, USA.

xinghua@pitt.edu.

## Question 55: How can we systematically reveal the immune evasion mechanism exploited by each tumor and utilize such information to guide targeted therapy to restore immune sensitivity?

### Background and implication

A cancer cell often hosts a fair number of nonsynonymous mutations. When mutant proteins are presented on cell surface by major histocompatibility complex, they may be neo-antigens recognizable by the host immune system, which can result in the elimination of cancer cells by the immune system. Thus, cancer cells expressing neo-antigens are constantly under the immune surveillance stress, and each solid tumor must have successfully escaped the surveillance through certain mechanisms. The success of immune-checkpoint protein inhibitors [12, 13] in restoring immune response against cancer cells provides strong evidence of vulnerability of cancer cells and existence of immune evasion mechanism exploited by tumors. It is hypothesized that tumors acquire such capability through Darwin evolution of cancer cells [14], likely through acquiring genomic alterations that endow the tumor with such capability. Recent work by Spranger et al. [15] indicate that pathway aberrations intrinsic to cancer cells can modulate immune environment of tumors. It is therefore important for us to understand the immune evasion mechanisms of individual tumors by systematically revealing the immune evasion mechanism exploited by each tumor. This knowledge would be very useful for guiding targeted therapy to restore immune sensitivity. We believe solving this issue requires novel computational methods that can infer the causal relationship between somatic genome alterations and changed immune environment, which can be measured as molecular phenotypes reflective of immune environment of a tumor; it also requires systems biology approaches to investigate how different cells interact in tumor microenvironment.

#### Submitter

Xinghua Lu.

#### Affiliation and email

Department of Biomedical Informatics, University of Pittsburgh, Pittsburgh, PA, USA.

xinghua@pitt.edu.

## Question 56: Can the nasopharyngeal carcinoma (NPC) patients with sarcomatoid carcinoma (SC) subtype benefit from more specific targeted therapy?

### Background and implication

The currently used World Health Organization (WHO) histopathological classification of NPC conveys little clinical relevance in terms of prognosis prediction. Recently, we have proposed a new histopathologic classification of NPC based on the morphologic features and cell differentiation of tumors [16]. In this classification, the 5-year overall survival (OS) rates for NPC patients who were diagnosed with epithelial carcinoma (EC; accounting for 61.5% of all NPC cases), mixed sarcomatoid-epithelial carcinoma (MSEC; accounting for 20.7%), sarcomatoid carcinoma (SC 13.6%), and squamous cell carcinoma (SCC 4.2%) were 79.4, 70.5, 59.6, and 42.6%, respectively (*P* < 0.001). Obviously, the NPC patients with SC have a lower 5-year OS rate than the patients with EC (59.6% vs. 79.4%). Our further analyses found that the addition of platinum-based chemotherapy to the standard radiotherapy could significantly benefit the NPC patients with EC by prolonging their OS, but could not benefit the patients with SC. In other words, SC is not sensitive to platinum-based chemotherapy and therefore should be treated differently. Many spindle tumor cells present in SC, suggesting their close relationship with epithelial–mesenchymal transition (EMT) process and more aggressive biological behavior. The genomic landscape of this subtype remains unclear. Our understanding of the underlying genomic changes of SC would make future targeted therapy possible and subsequently a better treatment outcome in the era of precision medicine.

#### Submitters

Hai-Yun Wang and Jian-Yong Shao.

#### Affiliation and emails

Department of molecular diagnostics, Sun Yat-sen University Cancer Center, Guangzhou, China.

wanghaiy@sysucc.org.cn; shaojy@sysucc.org.cn.

## References

[1] Qian CN, Zhang W, Xu RH (2016). Defeating cancer: the 150 most important questions in cancer research and clinical oncology. Chin J Cancer.

[2] Wee JT, Poh SS (2017). The most important questions in cancer research and clinical oncology. Question 1. Could the vertical transmission of human papilloma virus (HPV) infection account for the cause, characteristics, and epidemiology of HPV-positive oropharyngeal carcinoma, non-smoking East Asian female lung adenocarcinoma, and/or East Asian triple-negative breast carcinoma?. Chin J Cancer.

[3] Venniyoor A (2017). The most important questions in cancer research and clinical oncology—question 2–5. Obesity-related cancers: more questions than answers. Chin J Cancer.

[4] Chinese Journal of Cancer (2017). The 150 most important questions in cancer research and clinical oncology series: questions 6–14. Chin J Cancer.

[5] Chinese Journal of Cancer (2017). The 150 most important questions in cancer research and clinical oncology series: questions 15–24. Chin J Cancer.

[6] Chinese Journal of Cancer (2017). The 150 most important questions in cancer research and clinical oncology series: questions 25–30. Chin J Cancer.

[7] Chinese Journal of Cancer (2017). The 150 most important questions in cancer research and clinical oncology series: questions 31–39. Chin J Cancer.

[8] Chinese Journal of Cancer (2017). The 150 most important questions in cancer research and clinical oncology series: questions 40–49. Chin J Cancer.

[9] Steeg PS (2016). Targeting metastasis. Nat Rev Cancer.

[10] Kandoth C, McLellan MD, Vandin F, Ye K, Niu B, Lu C (2013). Mutational landscape and significance across 12 major cancer types. Nature.

[11] Lawrence MS, Stojanov P, Mermel CH, Robinson JT, Garraway LA, Golub TR (2014). Discovery and saturation analysis of cancer genes across 21 tumour types. Nature.

[12] Leach DR, Krummel MF, Allison JP (1996). Enhancement of antitumor immunity by CTLA-4 blockade. Science.

[13] Mueller KL (2015). Cancer immunology and immunotherapy. Realizing the promise. Introduction. Science.

[14] Kim R, Emi M, Tanabe K (2007). Cancer immunoediting from immune surveillance to immune escape. Immunology.

[15] Spranger S, Bao R, Gajewski TF (2015). Melanoma-intrinsic beta-catenin signalling prevents anti-tumour immunity. Nature.

[16] Wang HY, Chang YL, To KF, Hwang JS, Mai HQ, Feng YF (2016). A new prognostic histopathologic classification of nasopharyngeal carcinoma. Chin J Cancer.

